# Retroperitoneal Sarcoma Requiring Abdominal Aortic Replacement With Long-Term Survival: A Case Report

**DOI:** 10.7759/cureus.60198

**Published:** 2024-05-13

**Authors:** Yuta Inoue, Yukio Umeda, Shohei Mitta, Yukihiro Matsuno, Yoshio Mori

**Affiliations:** 1 Cardiovascular and Thoracic Surgery, Gifu Prefectural General Medical Center, Gifu, JPN

**Keywords:** long-term survival, complete resection, abdominal aortic replacement, combined abdominal aortic resection, retroperitoneal sarcoma

## Abstract

Retroperitoneal sarcoma (RPS) is a rare disease. RPS invading the abdominal aorta is exceedingly rare and has a poor prognosis. There have been scattered cases of RPS treated with combined abdominal aortic replacement. However, the average survival time for these cases was only 8 months, with a 2-year survival rate of 21%, indicating a poor prognosis. In this case study, a 44-year-old man presented to our hospital complaining of abdominal pain. Multiple imaging findings suggested a retroperitoneal mass that was diagnosed as a malignant tumor. The patient underwent tumor resection with abdominal aortic replacement due to an RPS tumor invading the abdominal aorta. The histopathological grade was determined to be grade 3, the most malignant grade tumor, according to the Fédération Nationale des Centres de Lutte Contre le Cancer grading system. Postoperative chemotherapy with doxorubicin and ifosfamide was administered for five cycles. The patient has been alive for over 8 years after the operation without any recurrence. This case presents a long-term survival of RPS requiring abdominal aortic replacement.

## Introduction

Retroperitoneal sarcoma (RPS) is a rare malignant tumor [1]. Instances involving the abdominal aorta are particularly infrequent and typically associated with an unfavorable outcome. Although there are reports of RPS invading the abdominal aorta which was treated with the abdominal aortic replacement [2], the data suggest limited postoperative survival, averaging only eight months, alongside a two-year survival probability of approximately 21% [3]. This underscores the generally poor prognosis of such cases.

In this article, we detail a case of RPS that necessitated abdominal aortic replacement and resulted in an exceptional postoperative survival for over 8 years.

## Case presentation

**Figure 1:**
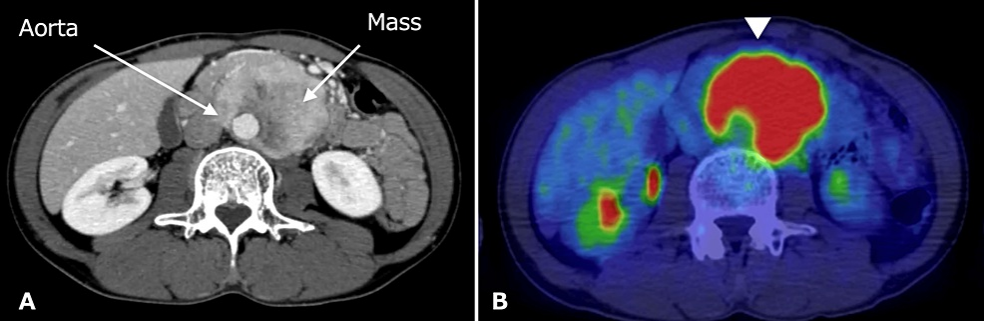
Preoperative CT findings Contrast-enhanced CT revealed a retroperitoneal mass of about 70 millimeters surrounding the abdominal aorta (A). PET scan showed a significant accumulation of the abdominal mass (arrowhead) (B).

A 44-year-old man was referred to our hospital for assessment of abdominal pain. On abdominal ultrasound, an abdominal mass surrounding the aorta was detected. Contrast-enhanced computed tomography (CT) revealed a retroperitoneal mass of about 70 millimeters surrounding the abdominal aorta, which is enhanced from the edges in the early phase, with gradual internal dyeing (Figure 1A). Fluorodeoxyglucose-position emission tomography (PET) scan showed a significant accumulation of the abdominal mass (Figure 1B).

**Figure 2:**
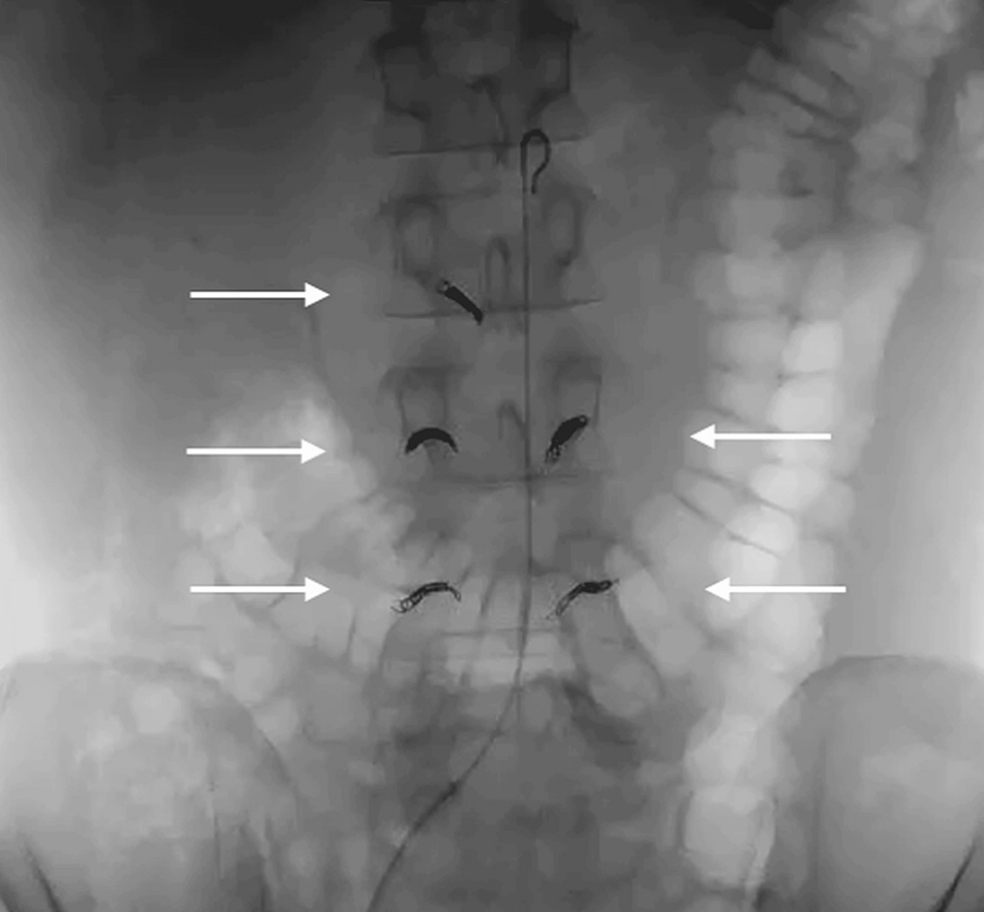
Preoperative embolization of lumbar arteries In order to control surgical bleeding, embolization of the right second, bilateral third, and fourth lumbar arteries was performed via interventional radiology (arrows) before the operation.

Laboratory assessment of tumor markers such as SCC, CA19-9, and CA125 were not positive. The mass was considered to be a malignancy and we performed a tumor resection with abdominal aortic replacement. In order to control surgical bleeding, embolization of the right second, bilateral third, and fourth lumbar arteries was performed via interventional radiology before the operation (Figure 2).

**Figure 3:**
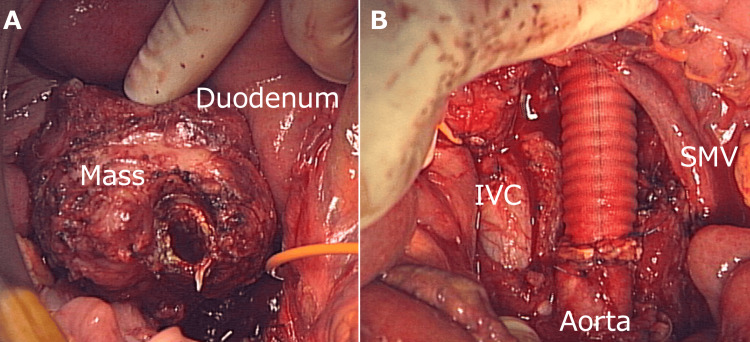
Operation; tumor resection with abdominal aortic replacement The patient underwent tumor resection with abdominal aortic replacement using a straight graft (A, B). (IVC,  Inferior Vena Cava; SMV, Superior Mesenteric Vein)

The patient underwent tumor resection with abdominal aortic replacement. Although the tumor was firmly adhered to the pancreatic head, it was successfully detached without any invasion of the duodenum or peritoneal dissemination. The tumor was completely resected grossly with the abdominal aorta after separately clamping the infrarenal and terminal aorta. The procedure involved abdominal aortic replacement using a straight artificial tube graft (Figure 3A, 3B). We did not reconstruct the inferior mesenteric artery because its bifurcation was occluded by the tumor.

**Figure 4:**
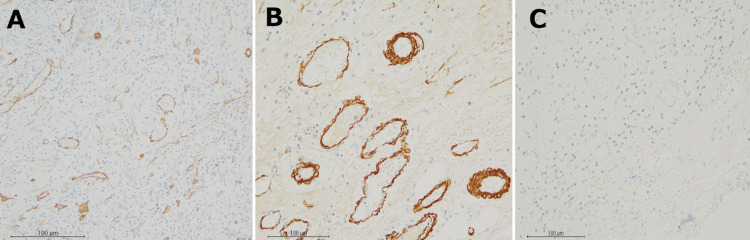
Histological findings The retroperitoneal mass demonstrated spindle-shaped or pleomorphic tumor cells with eosinophilic spores proliferating in a full and diffuse fashion. Immunohistochemically, positive results for alpha-smooth muscle actin (α-SMA) (A), HHF-35 (B), and negative for Desmin (C).

Histopathological examination of the retroperitoneal mass demonstrated spindle-shaped or pleomorphic tumor cells with eosinophilic spores proliferating in a full and diffuse fashion. Inflammatory cell infiltrations by plasma cells and lymphocytes were prominent in the background, accompanied by fibrous stromal hyperplasia. Immunohistochemically, the tumor cell showed positive results for alpha-smooth muscle actin (α-SMA) (Figure 4A, x10 objective), HHF-35 (Figure 4B, x40 objective), calponin, and negative for desmin (Figure 4C, x40 objective), SMMS-1, myogenin, ALK-1, CD34, CD31, CD45, CD68, D2-40, C-kit, β-catenin, S100, Neurofilament, p16, and MDM2 gene amplification.

**Figure 5:**
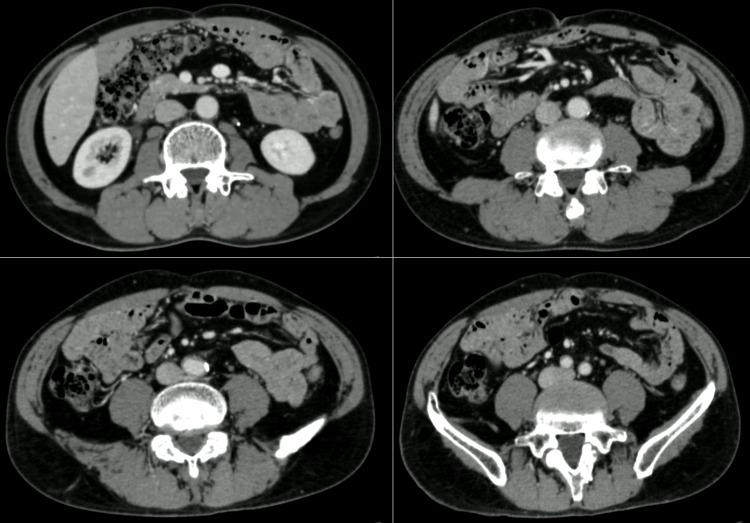
Contrast-enhanced CT at 8 years after operation There is no obvious local recurrence or distant metastasis.

Thus the tumor was considered to be an RPS. Additionally, the tumor cells invaded the abdominal aorta. The histological grade of the soft tissue sarcoma was classified as grade 3 according to the Fédération Nationale des Centres de Lutte Contre le Cancer (FNCLCC) grading system [4]. The patient was discharged without any complications on the 17th day after the operation. Following the surgery, the patient underwent five cycles of postoperative chemotherapy with doxorubicin and ifosfamide. Currently, the patient has survived for over 8 years since the operation without any signs of recurrence according to contrast-enhanced CT (Figure 5).

## Discussion

The FNCLCC grading system is used as the histopathologic classification of soft tissue tumors proposed by the French Federation of Cancer Centers Sarcoma Group [4]. In this case, the tumor differentiation score was 3, the mitosis count was 2, and the tumor necrosis score was 1, resulting in a total score of 6. This corresponds to Grade 3, which is the true Malignant Tumor. According to the FNCLCC grading system, the 5-year recurrence-free survival rate for Grade 1 patients is reported to be more than 90%, compared to about 40% for Grade 3 patients [4].

RPS invading the aorta is exceedingly rare [5]. Schwarzbach et al. retrospectively evaluated 141 patients with RPS, but only five cases underwent aortic replacement [6]. Furthermore, there are few reports of long-term survival in patients with RPS who required combined aortic replacement. Cananzi et al. reported that the average survival time for RPS requiring abdominal aortic reconstruction was 8 months, with a 2-year survival rate of 21%, indicating a poor prognosis [5]. In contrast to this report, despite the poor prognosis based on the FNCLCC grading, the patient has survived for over 8 years after surgery without recurrence. Two major factors may have contributed to this favorable outcome.

One is the complete resection. Quildrian et al. reported that complete surgical resection in RPS has a significant impact on overall survival and local recurrence [2]. Complete surgical resection is the mainstay of treatment in RPS and local recurrence rates are closely related to the status of surgical margins [7]. Major blood vessel involvement is challenging for surgeons because vascular resection and immediate reconstruction are mandatory to obtain complete resection, however, it might be necessary to perform en-bloc resections in case of invasion of either surrounding tissues or adjacent organs [8, 9]. In this case, it is suggested that the patient achieved complete gross resection, indicating that this treatment strategy may have been beneficial in improving the patient's prognosis.

Another factor is postoperative chemotherapy. The administration of perioperative chemotherapy should always be evaluated by a multidisciplinary team on an individualized basis, as no definitive evidence establishing the role of multimodal treatment in RPS has been available so far [10, 11]. Surgery is the cornerstone of RPS treatment but adjuvant treatment may be of benefit in selected patients [5]. Another report notes that the administration of chemotherapy in a preoperative setting may allow not only the shrinkage of the primary tumor but it may also inhibit eventual occult distant micrometastatic lesions [12]. Recently, a retrospective multi-institutional study [13] analyzed the responsiveness of RPS to neo-adjuvant chemotherapy, suggesting some benefit, especially in some histologic subtypes. Chemotherapy may be considered for chemotherapy-sensitive tumors, such as synovial sarcoma and myxoid liposarcoma, and for tumors at high risk of distant metastasis, such as large-sized dedifferentiated liposarcoma [14]. However, all existing evidence on the use of chemotherapy in RPS is derived from retrospective series or clinical trials on extremity soft-tissue sarcomas, which has included only a small number of patients with RPS. As a result, at present, chemotherapy cannot be routinely recommended for preoperative treatment of resectable RPS. In our case, postoperative chemotherapy with ifosfamide and doxorubicin was administered due to the large size and high grade of malignancy, which may also be an effective strategy. We have to continue discussing the possibility of using these systemic therapies perioperatively.

A limitation exists in that it is unclear if the tumor is a leiomyosarcoma or a myofibroblastic sarcoma according to the pathological findings. It is also unclear how effective was the postoperative chemotherapy. 

## Conclusions

We presented a case of RPS which required abdominal aortic replacement, resulting in long-term survival. The effectiveness of perioperative chemotherapy and radiotherapy for RPS is still unclear due to the rarity of the disease. Further establishment of multidisciplinary treatment strategies, including perioperative chemotherapy and radiation therapy, is needed to improve the survival rate of RPS with large vessel invasion.

## References

[1] Schmitz E, Nessim C (2022). Retroperitoneal sarcoma care in 2021. Cancers (Basel).

[2] Quildrian SD, Nardi WS, David M (2020). Resection of retroperitoneal soft-tissue sarcoma involving the abdominal aorta. BMJ Case Rep.

[3] Wortmann M, Alldinger I, Böckler D, Ulrich A, Hyhlik-Dürr A (2017). Vascular reconstruction after retroperitoneal and lower extremity sarcoma resection. Eur J Surg Oncol.

[4] Trojani M, Contesso G, Coindre JM (1984). Soft-tissue sarcomas of adults; study of pathological prognostic variables and definition of a histopathological grading system. Int J Cancer.

[5] Cananzi FC, Ruspi L, Fiore M, Sicoli F, Quagliuolo V, Gronchi A (2021). Major vascular resection in retroperitoneal sarcoma surgery. Surgery.

[6] Schwarzbach MH, Hormann Y, Hinz U (2006). Clinical results of surgery for retroperitoneal sarcoma with major blood vessel involvement. J Vasc Surg.

[7] Gundle KR, Kafchinski L, Gupta S (2018). Analysis of margin classification systems for assessing the risk of local recurrence after soft tissue sarcoma resection. J Clin Oncol.

[8] Quagliuolo V, Gronchi A (2020). Current Treatments of Retroperitoneal Sarcomas. Updates Surg.

[9] Radaelli S, Fiore M, Colombo C (2016). Vascular resection en-bloc with tumor removal and graft reconstruction is safe and effective in soft tissue sarcoma (STS) of the extremities and retroperitoneum. Surg Oncol.

[10] Haas RL, Gronchi A, van de Sande MA (2018). Perioperative management of extremity soft tissue sarcomas. J Clin Oncol.

[11] DeLaney T, Mullen JT, Wang D, Goldberg SI, Kirsch DG (2021). Preoperative radio- therapy for retroperitoneal sarcoma. Lancet Oncol.

[12] Gamboa AC, Gronchi A, Cardona K (2020). Soft-tissue sarcoma in adults: an update on the current state of histiotype-specific management in an era of personalized medicine. CA Cancer J Clin.

[13] Tseng WW, Barretta F, Conti L (2021). Defining the role of neoadjuvant systemic therapy in high-risk retroperitoneal sarcoma: a multi-institutional study from the Transatlantic Australasian Retroperitoneal Sarcoma Working Group. Cancer.

[14] Canter RJ, Qin LX, Maki RG, Brennan MF, Ladanyi M, Singer S (2008). A synovial sarcoma-specific preoperative nomogram supports a survival benefit to ifosfamide-based chemotherapy and improves risk stratification for patients. Clin Cancer Res.

